# Credibility attacks do not enhance the impact of deepfake warnings

**DOI:** 10.1038/s41598-026-64243-y

**Published:** 2026-08-05

**Authors:** Emily R. Spearing, Kaitlin Norgard, Simon Clark, Stephan Lewandowsky, Ullrich K. H. Ecker

**Affiliations:** 1 School of Psychological Science, University of Western, Perth, Australia; 2 School of Psychological Science, University of Bristol, Bristol, England; 3 Department of Psychology, University of Potsdam, Potsdam, Germany; 4 Public Policy Institute, University of Western, Perth, Australia

**Keywords:** Deepfakes, Misinformation, Warnings, Artificial intelligence, Credibility, Psychology, Psychology, Science, technology and society

## Abstract

Synthetic deepfake videos are a realistic form of misinformation, which appear to show someone saying or doing something they never did. The societal risks posed by such fakeries have motivated legislation mandating the labelling of AI-generated content, but initial evidence suggests that even explicit warnings are insufficient to eliminate deepfake influences on viewer perceptions. Across two experiments (total *N =* 2,500), we examined the impact of a deepfake video in which a witness makes a criminal accusation, and we tested whether undermining the credibility of the witness could enhance the effect of a specific warning flagging the video as a deepfake. Two methods for reducing witness credibility were tested: presenting the witness with a foreign (vs. native) accent and explicitly introducing them as an untrustworthy (vs. trustworthy) character. Deepfake exposure significantly increased guilt judgements concerning the person being accused in the video; a deepfake warning only partially reduced this impact. When an explicit witness discreditation was added to the warning, effectiveness remained unchanged. Findings demonstrate the pervasive impact of known deepfakes, highlighting the need to combine or develop new interventions to combat their influence.

**Supplementary Information:**

The online version contains supplementary material available at https://doi.org/10.1038/s41598-026-64243-y.

## Introduction

Digitally manipulated content has long been recognised as a persuasive source of misinformation^1,2^. Until recently, however, its proliferation was limited by the substantial technical expertise required to produce convincing synthetic media^3^. The rapid advancement and growing accessibility of artificial intelligence (AI) technology means that almost anyone can now create realistic audiovisual content, including convincing deepfake videos that appear to show individuals saying or doing something that they never said or did. Such fakeries can be designed to maximise salience, believability, and shareability, capitalising on pre-existing biases towards negative and novel narratives^4,5^ and are often indistinguishable from real videos^6–9^. In the current study, we examined whether the impact of a deepfake accusatory video—in which an alleged witness accused another (undepicted) person of a crime—can be reduced by a specific warning labelling the video as fake. To elucidate the cognitive processing of warnings, we also examined whether they are more effective when they are accompanied by additional factors that reduce perceived credibility of the depicted witness.

While deepfakes present opportunities for entertainment and education^10–12^, they also pose significant threats to society; they can be used to manipulate, threaten, and defraud individuals, influence democratic processes, and undermine criminal justice through the creation of counterfeit evidence^13–18^. Research shows, for example, that deepfakes can damage perceptions of targeted politicians^19^, produce misconceptions in important domains (e.g., health)^20^, and increase the credibility of accompanying textual misinformation^21^.

The potential for deepfakes to be used for nefarious purposes—combined with the finding that people typically struggle to discern them from real videos^6,7^—has contributed to legislation intended to increase the transparency of AI-generated content and reduce potential harms^22,23^. The European Union’s AI Act, for example, requires the labelling of artificially generated content, and prohibits the use of AI systems for manipulation, deception, and exploitation^24^. Additionally, many social media platforms have used third party fact-checkers and increasingly rely on community members to flag misleading and AI-generated content^25,26^. Such countermeasures are intended to empower individuals to identify unreputable sources and disregard misleading content. Initial evidence, however, suggests that warnings are likely insufficient for combatting the influence of AI-generated content and may instead produce general scepticism^27–31^. General warnings that highlight the existence of misinformation or synthetic content, for example, have been found to offer little to no benefit for detecting deepfakes and often reduce trust in all content^32–36^. While fostering scepticism can be beneficial for developing resistance against misinformation, increasing people’s willingness to disregard reliable information could contribute to cynicism in public debate, undermining efforts to maintain public confidence in authentic content, and enable individuals to write off credible evidence as supposed deepfakes^5,8,37^.

Specific warnings that flag pieces of inaccurate or AI-generated content are only somewhat more effective. While such labels offer some benefit for reducing the perceived credibility of misinformation and the engagement it receives on social media^32,38–40^, research suggests that they seldom eliminate its influence on cognition entirely^41^. One study, for example, found that exposure to a deepfake confession increased ratings of the accused’s guilt; this impact was significantly reduced by a warning stating the video was a deepfake, but warned participants nevertheless remained significantly influenced by the confession^42^. These findings are perhaps not surprising in light of myriad studies showing that misinformation frequently continues to influence cognition even after people have received a clear and credible correction (i.e., the “continued influence effect”)^43–46^.

The finding that warnings seldom eliminate the impact of misinformation and sometimes produce general scepticism highlights the importance of developing new countermeasures to combat misleading media. One factor that is well known to substantially affect belief in true and false information^47,48^ and correction effectiveness^49–51^ is source credibility. News items are perceived as more truthful, for example, when they come from a reputable source than when they come from a fictional source or one with a poor reputation^52,53^. Accordingly, discrediting an unreputable source—such as by pointing out a conflict of interest or a track record of spreading false information—can reduce the impact of misinformation and increase the effectiveness of corrective information^54,55^. One study, for instance, found that while warning participants that generative AI systems can make errors was insufficient on its own to reduce the impact of a misleading AI-generated text on reasoning, it improved the effectiveness of a retroactive correction, with the combined intervention eliminating the article’s impact^29^. It has also been found that corrective information can reduce the credibility of a misinformation source, and that these effects are larger when participants are also given explicit information undermining the source’s credibility (i.e., suggesting that they lied to get revenge)^56^. Hitherto, however, no research—to the best of our knowledge—has examined whether undermining the credibility of a *fake* source can reduce the impact of deepfakes or increase the effectiveness of warnings designed to combat their impacts. The present study therefore examined whether a pre-emptive, specific warning could reduce the impact of a deepfake accusatory video, and whether additional factors undermining source credibility—specifically, the depicted witness’s credibility—could bolster those effects.

## Experiment 1

One factor that is well-known to affect the perceived credibility of an information source is accent. A large body of research demonstrates that individuals with standard, native accents are often perceived more favourably than those with non-standard, foreign accents—a phenomenon known as accent bias^57,58^. This bias appears to operate across key dimensions of credibility: warmth/trustworthiness and competence/expertise^59^. Standard-accented individuals are, for instance, perceived as both warmer and more competent in hiring interviews^60,61^ and as providing more truthful, credible, and accurate eyewitness testimony^62^ than foreign-accented individuals. Accent bias has been replicated across various accents and using computer-generated avatars in place of real, visible people^63^. It is at least partly driven by in-group/out-group dynamics: native accents often signal in-group membership, whereas foreign accents often mark the speaker as an outgroup member, triggering less favourable social evaluations^64–66^. Accent effects are further shaped by socio-linguistic stereotypes^61^; they are particularly pronounced when the foreign accent is associated with an already stigmatised group, with “high status” accents (associated with higher class, wealth, and education) being rated more favourably than “low status” accents. Accent effects are also stronger for female speakers^61,67^. These findings raise the possibility that the impact of deepfake content might be attenuated when it is delivered by an ostensibly less credible, foreign-accented speaker, and that this might serve as a catalyst for a warning intervention to be more effective.

Experiment 1 therefore aimed to examine two approaches to experimentally manipulating the perceived credibility of deepfakes and their influence on perceptions: namely, the use of (1) a pre-emptive, specific warning identifying the video as fake, and (2) a foreign (Indian) accent, compared to a standard (British) accent. Participants read an incident briefing outlining the events of a fictional hit-and-run accident and were then randomly allocated to watch one of two deepfakes in which an alleged witness names a (fictional) local politician as the culprit, speaking in either a British or Indian accent. Depending on condition, the video was preceded by either a warning that identified the video as a likely deepfake or no warning. We measured perceptions of the politician’s perceived guilt through a combination of rating and open-ended questions, as well as the perceived fakeness of the deepfake. In an additional control condition, only the incident briefing was presented. Materials and data for both experiments are available at: https://osf.io/5jx3h/overview?view_only=84a2f527811a4e15bf1c73f1fda1df99.

We predicted that exposure to the deepfake video would increase perceptions of guilt (H1), even when participants received a warning regarding its fakeness (H2); it was expected, however, that the warning would reduce the influence of the deepfake on guilt perceptions to some extent (H3). We further hypothesised that guilt ratings would be lower (H4) and that the warning would be more effective (H5) in the Indian-accent deepfake condition, compared to the British-accent deepfake condition. Finally, we expected that the deepfake’s perceived reliability would decrease (H6) and its perceived fakeness would increase (H7) when presented with (a) the warning (vs. no warning) and (b) an Indian accent (vs. a British accent).

### Method

Experiment 1 used a 2 (warning: no warning, warning) × 2 (accent: British, Indian) between-subjects design, with an additional control group that did not see a deepfake. This research was approved by the Human Research Ethics Office of the University of Western Australia (ethics approval RA/4/20/6423). Informed consent was obtained from all participants, and all methods were performed in accordance with the relevant guidelines and regulations.

#### Participants

We conducted a simulation-based power analysis for a 2 × 2 ANOVA in R 4.4.1, based on the outcomes of comparable prior work (i.e., condition means for perceived guilt and the standard deviation of 1.6 for a deepfake video in Clark & Lewandowsky, 2026; Experiment 3). Statistical power was established for various sample sizes by calculating the proportion of statistically significant (*p* < .05) effects from 1,000 simulations. The results indicated that 250 participants per between-subjects condition would be sufficient to detect a small interaction effect (*f* = 0.1) with 80% power. We thus collected data from *N =* 1250 UK-based participants via Prolific (www.prolific.co), who had a platform-approval rating of at least 99%, including 595 men, 652 women, 2 agender, and 1 non-binary participant (*M*_*age*_ = 44.58 years; *SD* = 13.29, range = 19–81). Participants rated their English proficiency as “excellent” (*n =* 1147, 91.76%), “very good” (*n =* 91, 7.28%), or “good” (*n =* 12, 0.96%); none reported “fair” or “poor” proficiency. All participants were included in the analyses.

#### Materials

##### Incident briefing

The 399-word incident briefing comprised four statements, which outlined the events of a fictional car crash and the testimonies of relevant stakeholders. The first statement provided a description of the incident (“Last Monday at 6:15am, a hit-and-run car crash occurred on the intersection of Richard Street and Victoria Lane in Clifton”) and the victim’s injuries (“The incident resulted in serious harm to Brown, who suffered from fractured ribs, a dislocated shoulder, and a head injury”). This was followed by: (1) a witness account of the incident and emergency response (e.g., “When I turned onto Richard Street I saw there had been a crash and a white sedan was just speeding away”), (2) a victim-impact statement outlining the harm caused to the victim and her family (“Simone’s injuries are quite serious … I’ve been looking after the kids. I take them to visit her, but they are so young, it’s hard for them to see her like that”), and (3) a public statement by a local politician, Christian Whittaker, commenting on the quality of Clifton’s roads (e.g., “It must be said that there have just been too many incidents on this road. It’s dangerous!”) and stating that the perpetrator would be “caught and punished appropriately”. The primary purpose of the incident briefing was to provide participants with the information needed to make judgements about the case, particularly in the absence of the deepfake accusation video in the control condition. The full incident briefing is provided in the Supplementary Materials.

##### Deepfake videos

Two short deepfake videos (53–54 s) were created using the generative AI software tool Synthesia (www.synthesia.io). To this end, an AI model was trained on real videos of a mixed-race female actor, then prompted to produce two videos that appeared to depict the woman speaking in a British or Indian accent; the two voice models were selected to be similar in tone, pitch, and pacing, while also matching the age and appearance of the actress. A mixed-race woman was selected to play this role to make both accents plausible, and because of the gendered nature of accent bias^61,67^. The videos were generated using the same script (see the Supplementary Materials) and appear to show a woman talking in a podcast interview; she claims that she witnessed the hit-and-run accident in her local area and accuses politician Christian Whittaker as the driver-at-fault (“He drove right past me and I saw in, clear as day. It was Christian Whittaker. Yeah, the politician. I recognised him from those posters”).

##### Warning

In the warning conditions, participants were given a specific warning (“WARNING: the following video has been flagged as a deepfake, and therefore the content is likely to be false.”), followed by a brief definition of the term ‘deepfake’ (“A deepfake is a highly realistic AI-generated video that appears to depict someone doing or saying something that never actually happened”). The warning was designed to be clear but realistic, using probabilistic rather than deterministic phrasing.

##### Questionnaire

Participants answered three open-ended questions, which prompted them to summarise their understanding of the incident (e.g., “Please summarise in 2–3 sentences what you think happened in Clifton on Monday morning”); depending on the question, there was a requirement to provide at least 5–50 characters. Following prior work^42^, participants then answered eight questions on a 7-point Likert scale ranging from − 3 (*Definitely not*) to 3 (*Definitely yes*); the midpoint (0) was labelled “*Impossible to say*”, providing a normatively appropriate response when participants judged that there was insufficient reliable evidence to make a judgement. The scale was intended to measure the extent to which participants’ responses were consistent with the accusatory deepfake—rather than the extent to which they were accurate—with ‘*Definitely yes*’ reflecting complete belief in the accusation and ‘*Definitely not*’ reflecting complete disbelief. Given that the case was fictional, there is no ground truth regarding Christian Whittaker’s involvement in the incident, and thus no objective basis to determine the accuracy of participants’ responses.

The first five questions served as distractors designed to reduce demand characteristics (e.g., “Have the police been effective in their handling of the situation?”), while the remaining three questions assessed perceptions of Christian Whittaker’s guilt; participants who gave a rating of 1 (Maybe yes) or higher to the final question (i.e., “Is Christian Whittaker guilty of a crime?”) were also asked to describe the reasons for their answer. Participants in the experimental conditions also answered two questions assessing their perceptions of video fakeness (i.e., “Do you think the video was a deepfake?”) and reliability (i.e., “Was the information provided by this video reliable?”) using the same 7-point scale. Each rating question was presented for a minimum of 2 s.

#### Procedure

Participants viewed an ethics-approved information form, gave informed consent by ticking a box, provided demographic information (i.e., age, gender, ethnicity), and were randomly assigned to one of the five conditions (with the constraint of maintaining approximately equal cell sizes). All participants were then presented with the incident briefing. Participants in the experimental conditions were then told they would see “a snippet from a local community podcast” that “had surfaced on social media”; participants in the warning conditions were also given the warning, which was presented immediately before the video for a minimum of 5 s. Control participants did not see a video after the incident briefing and instead proceeded to the next part of the experiment. Next, all participants completed the questionnaire. Participants were then debriefed and received £1.20 for completing the experiment, which took approximately 8 min.

#### Data coding

Responses to open-ended questions were coded as 0, 0.5, or 1, based on whether they indicated that Christian Whittaker was guilty and thus the driver-at-fault in the incident. Specifically, responses were coded as 1 when they indicated Christian Whittaker’s involvement in the incident (e.g., “The driver who caused the crash, who happens to be a politician, fled the scene”), as 0.5 when they expressed doubt or uncertainty about Whittaker’s involvement (e.g., “They still don’t know who the driver was … There are claims it is a local politician but this cannot be confirmed”), and as 0 when they did not mention Christian Whittaker. K.N. coded all responses and E.S. independently coded responses from *n =* 125 participants (10%), blinded to condition. Inter-rater reliability was found to be excellent, Krippendorff’s α = 0.97. Discrepancies were resolved by a third coder (U.E.).

### Results

#### Perceptions of Guilt

**Fig. 1:**
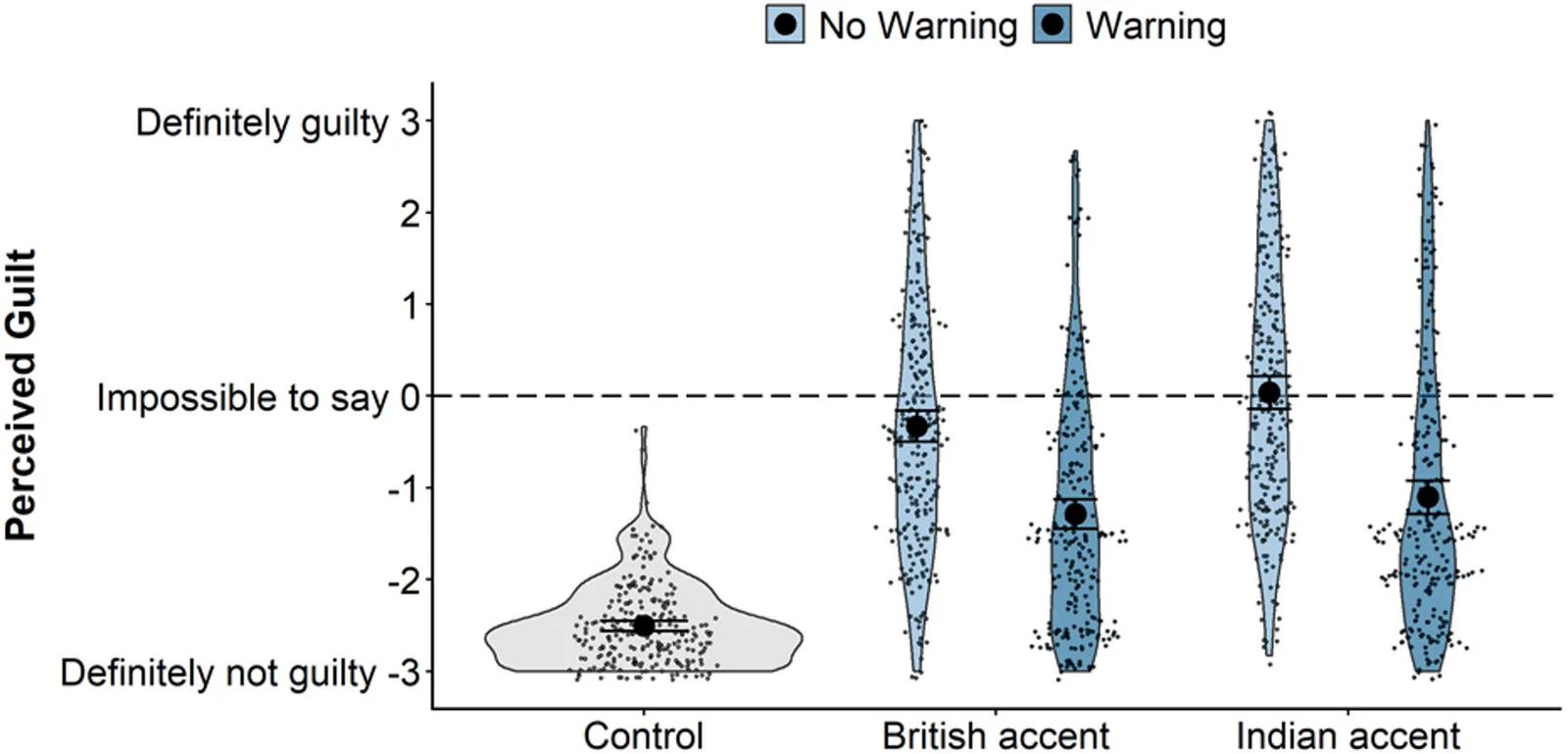
Mean Guilt Scores Across Conditions in Experiment 1. *Note.* Error bars denote 95% CIs. Data points indicate participant means.

To examine the impact of deepfake exposure on perceptions, we first calculated a guilt composite score by averaging responses to the three open-ended questions—transformed onto a -3 to 3 scale by replacing codes of 0, 0.5, and 1 with scores of -3, 0, and 3, respectively—and the three rating questions regarding Christian Whittaker’s guilt (α = 0.84), with higher values indicating greater reliance on the deepfake. We then conducted a one-way ANOVA on these scores with condition as the independent variable. Analyses were conducted using the *ez* package in R, and planned contrasts were conducted using the *emmeans* package. There was a significant effect of condition, *F*(4, 1245) = 152.25, *p* < .001, η_p_² = 0.328 (Fig. 1). Guilt scores were significantly higher when participants viewed the deepfake than when they did not, supporting H1; this effect was significant regardless of whether participants received the warning, supporting H2.

To assess the impacts of warning and accent directly, we conducted a two-way ANOVA on guilt scores (see Table 1), excluding control (*n*_control_ *=* 246, leaving *N =* 1004). There was a significant effect of warning, such that guilt scores were significantly lower for warned participants, supporting H3. There was also a small but significant effect of accent. However, contrary to H4, the Indian-accent deepfake elicited higher guilt scores than the British-accent deepfake. The interaction between warning and accent was nonsignificant, suggesting that warnings were equally effective for the British-accent and Indian-accent deepfakes, so H5 was not supported.

**Table 1:** Results of 2 × 2 ANOVA on Perceived Guilt in Experiment 1.

Effect	F(1, 1000)	*p*	η_*p*_^2^
Warning	144.36	< 0.001	0.126
Accent	9.90	0.002	0.010
Warning × Accent	1.15	0.285	0.001

#### Perceptions of deepfake video

**Fig. 2:**
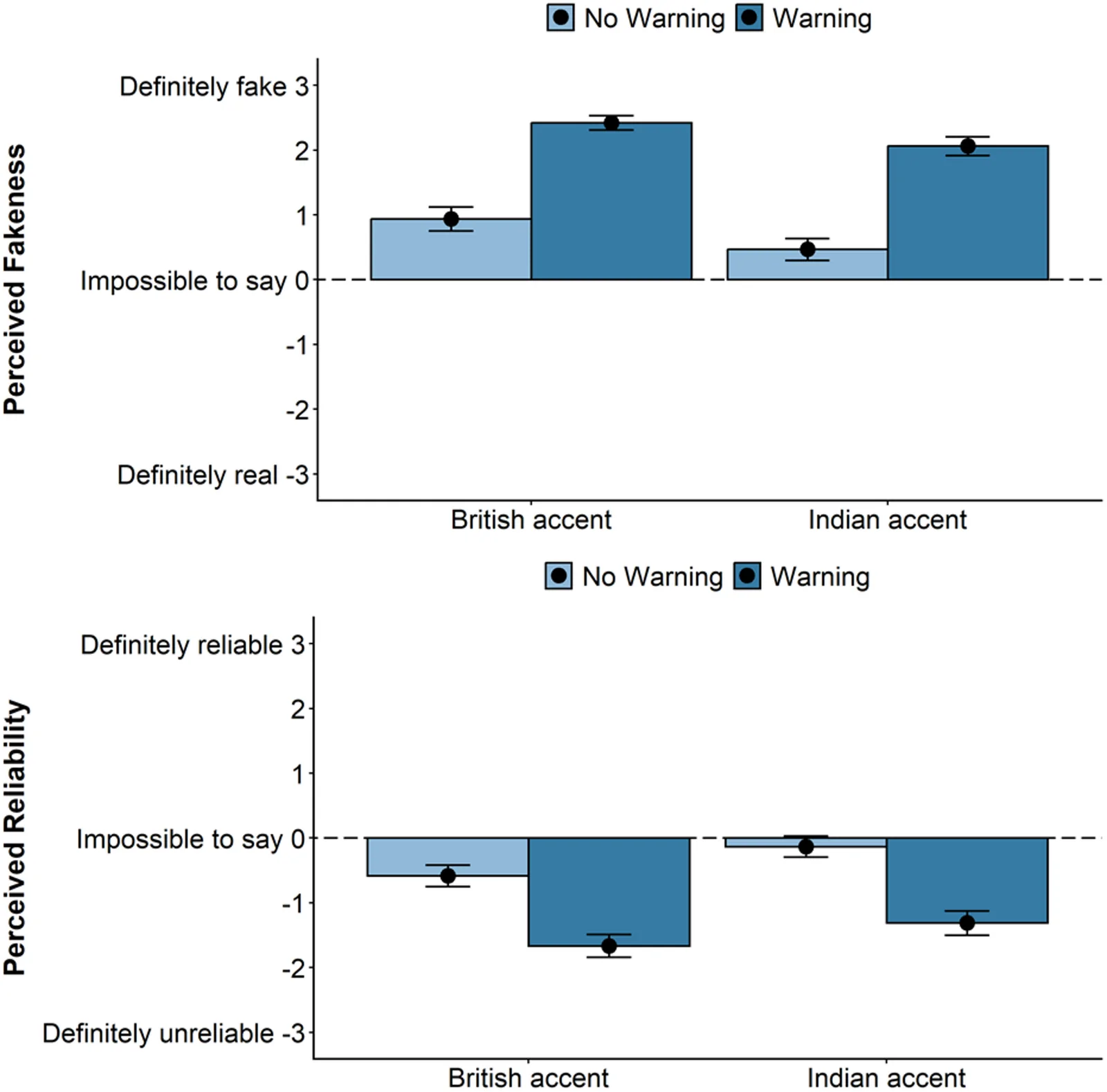
Mean Perceived Fakeness (top) and Reliability (bottom) of the Deepfake Video in *Experiment 1*. *Note.* Error bars denote 95% CIs.

To examine whether warning and accent influenced the perceived credibility of the deepfake, we conducted two-way ANOVAs separately for perceptions of fakeness and reliability (Table 2). Both revealed significant main effects of warning and accent and a nonsignificant interaction. Participants perceived the video as more likely to be a deepfake and as less reliable when (1) they were given a warning than when they were not, supporting H6a and H7a, and (2) when the witness appeared to speak with a British accent rather than an Indian accent, contrary to H6b and H7b (see Fig. 2).

**Table 2:** Results of 2 × 2 ANOVAs on perceived deepfake fakeness and reliability in Experiment 1.

Effect	F(1, 1000)	*p*	η_*p*_^2^
Fakeness			
Warning	378.05	< 0.001	0.274
Accent	27.23	< 0.001	0.027
Warning × Accent	0.50	0.481	0.000
Reliability			
Warning	162.09	< 0.001	0.139
Accent	20.50	< 0.001	0.020
Warning × Accent	0.29	0.588	0.000

### Discussion

In line with previous research^42^, Experiment 1 found that a specific warning reduced the perceived reliability of a deepfake accusatory video but only partially reduced its impact on guilt judgements. The warning was no more effective when combined with a foreign accent—designed to reduce perceived credibility of the deepfake accuser—than with a standard accent. In fact, the video produced greater perceptions of guilt and was perceived as more reliable and less likely to be a deepfake when it depicted the witness talking with a foreign accent, rather than a standard accent. The deepfake and warning effects will be more fully discussed after the results from Experiment 2 have been presented, so the following paragraph focuses on the accent findings.

In contrast to prior work showing that standard-accented individuals are perceived more favourably than foreign-accented individuals^60,67,68^, we found that UK participants perceived an accusation as more credible and gave higher guilt judgements when the video depicted an Indian-accented witness than when it depicted a British-accented witness. One possible explanation for this discrepancy is that most prior work has used authentic, human voices, whereas the current study used AI-generated audio, which may deviate from normal speech in detectable ways. Such deviations in deepfake voices may be easier to detect when they use a standard accent than when they use a more unfamiliar, foreign accent. Exploratory correlational analyses provided tentative support for this idea: in the absence of a warning, guilt judgements were negatively correlated with the perceived fakeness of the deepfake, but only when the witness spoke in a familiar British accent (*r =* -.24, *p* < .001) rather than an Indian accent (*r = -*.10, *p* = .138). Furthermore, prior research shows that people can detect deepfake speech with a standard accent with approximately 70% accuracy^69^, attend more closely to information presented in their own (standard) accent^70^, and are better at detecting deepfakes depicting someone who is demographically similar to themselves^71^. If the UK-based participants in the current study detected more deviations in the British-accented deepfake, then this might explain why it received lower credibility and higher fakeness ratings and had a smaller impact on guilt judgements than the Indian-accented deepfake. Taken together, these findings suggest that standard-accented deepfakes may be easier to detect and may thus have a smaller impact on cognition than foreign-accented deepfakes.

## Experiment 2

Experiment 1’s subtle attempt to experimentally manipulate source credibility via the witness’s accent produced an unexpected outcome: the Indian-accent deepfake elicited higher guilt judgements and was perceived as more reliable than the British-accent deepfake. Experiment 2 therefore examined whether providing explicit information about the witness’s credibility could be more effective at reducing the impact of a deepfake accusation and boost the effectiveness of a warning—an approach that is also more ecologically relevant. In the experimental conditions, participants read the incident briefing, then watched the British-accent deepfake. In contrast to Experiment 1, the video started with a voiceover that either undermined or bolstered the alleged witness’s credibility; the video was again preceded by either a warning regarding its fakeness or no warning. In the control condition, participants only read the incident briefing and did not see a video.

We predicted that exposure to a deepfake video would increase perceived guilt (H1) even when participants received a warning regarding its fakeness (H2), and that a warning would reduce (but not eliminate) its influence on perceptions (H3). We also hypothesised that guilt ratings would be reduced (H4) and that the warning would be more effective (H5) in the low source credibility condition than in the high source credibility condition. Finally, we expected that the deepfake’s perceived fakeness would increase (H6) and its perceived reliability would decrease (H7) when it was presented with (a) a warning (vs. no warning) or (b) explicit information undermining (vs. bolstering) source credibility.

### Method

Experiment 2 used a 2 (warning: no warning, warning) × 2 (source credibility: high, low) between-subjects design, with an additional control group that did not see a deepfake.

#### Participants

As in Experiment 1, we collected data from *N =* 1,250 UK-based participants via Prolific, who had a platform-approval rating of at least 99% and who had not taken part in Experiment 1, including 536 men, 710 women, 2 gender-fluid/non-binary participants, and 2 participants who did not disclose their gender (*M*_*age*_ *=* 43.82 years, *SD =* 13.61, range = 18–82). Most participants rated their English proficiency as excellent (*n* = 1174, 93.92%), very good (*n =* 70, 5.60%), while some reported good (*n =* 5, 0.4%) or fair (*n =* 1, 0.08%) proficiency. No participants reported “poor” proficiency. Experiment 1 participants were not allowed to participate in Experiment 2.

#### Materials

##### Deepfake videos

The British-accent video from Experiment 1 was edited to create two versions that each began with a voiceover introducing the alleged witness. Voiceovers were generated from scripts of 96–100 words using the AI voice-generator software (https://elevenlabs.io/) Elevenlabs and presented with rolling subtitles to aid participants’ comprehension. One version was designed to present the witness as highly credible and introduced her as “Dr Anjali Patel, a long-time member of the community and respected local GP”, while the other was designed to undermine her credibility as a witness and introduced her as “Anjalikumaraswamy Srinivasan, a local socialite with a known record of seeking publicity and sharing strong opinions on community issues, who also has a number of historic fraud convictions”. Voiceovers were designed to mimic third-party commentary (e.g., journalistic narration) that provides contextual information for evaluating source credibility after a video has been created and circulated (rather than how malicious actors might strategically leverage a speaker’s credibility when constructing deepfake content). To create clear separation between the voiceover and podcast content, the voiceover was presented as a distinct segment, accompanied by a short jingle and logo, prior to the podcast clip.

##### Questionnaire

The questionnaire was adapted from Experiment 1. Supplementary analyses of Experiment 1 data indicated that results using only rating-scale questions were comparable to the results based on all questions. Therefore, the open-ended questions were removed, and two additional rating questions were added. One served to provide an additional measure of the perceived guilt of Christian Whittaker (i.e., “Was Christian Whittaker the driver who fled the scene?”), and one assessed the perceived reliability of the voiceover (i.e., “Was the information about the podcast speaker provided in the video voiceover reliable?”) and was therefore not presented to control participants. All questions used the same 7-point scale as in Experiment 1.

#### Procedure

The procedure was identical to Experiment 1. Participants received £1.05 for completing the experiment, which took approximately 7 min.

### Results

#### Perceptions of Guilt

**Fig. 3:**
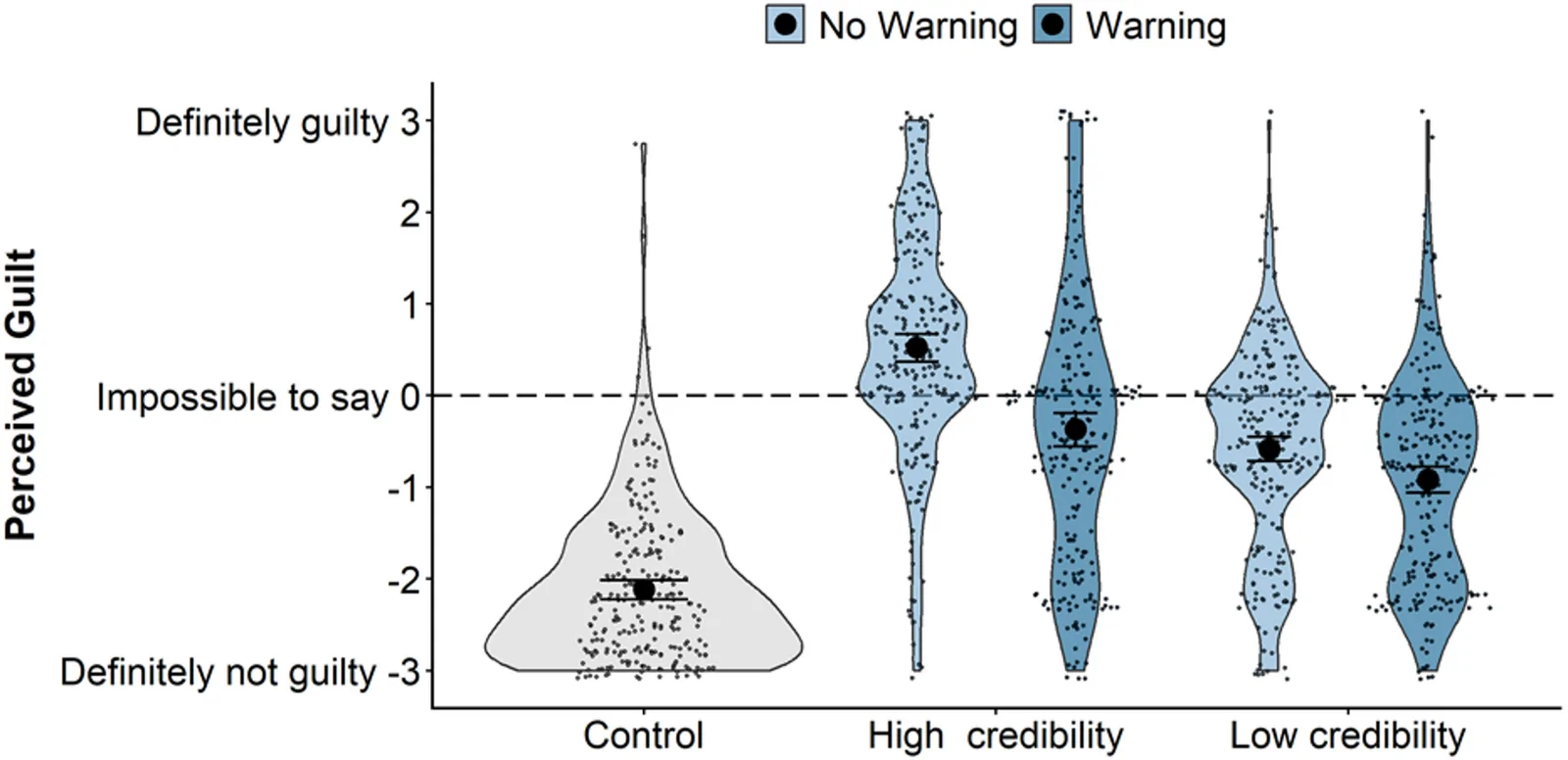
Mean guilt scores across conditions in Experiment 2. *Note.* Error bars denote 95% CIs. Data points indicate participant means.

To examine the impact of the deepfake video on perceived guilt, we first calculated a guilt composite score by averaging responses to the four rating questions regarding Christian Whittaker’s guilt (α = 0.90), then conducted a one-way ANOVA on those scores with condition as the independent variable. There was a significant effect of condition, *F*(4, 1245) = 172.91, *p* < .001, η_p_^2^ = 0.357 (Fig. 3). Consistent with Experiment 1, all experimental conditions produced significantly higher guilt ratings than control, all *F* ≥ 135.52, *p* < .001, η_p_^2^ ≥ 0.10, indicating that the video increased participants’ perceptions of guilt even when they were warned that it was a deepfake. Thus, H1 and H2 were supported.

Next, we conducted a two-way ANOVA on guilt ratings with warning and source credibility as independent variables (see Table 3), excluding control (*n*_control_ = 254, leaving *N =* 996). There were significant main effects of warning and source credibility; guilt scores were significantly reduced (1) when participants received the warning compared to when they did not, supporting H3, and (2) when the voiceover undermined source credibility compared to when it presented the witness as highly credible, in line with H4. These effects, however, were qualified by a significant interaction. The warning significantly reduced perceived guilt regardless of source credibility (high: *F*[1, 992] = 66.28, *p <* .001, η_p_^2^_=_ 0.064; low: *F*[1, 992] = 9.35, *p* = .002, η_p_^2^ = 0.010), but this reduction was significantly larger in the high source credibility condition than in the low source credibility condition, *F*(1, 992) = 12.98, *p* < .001, η_p_^2^ = 0.014. Thus, H5 was not supported.

**Table 3:** Results of 2 × 2 ANOVA on Perceived Guilt in Experiment 2.

Effect	F(1, 992)	*p*	η_*p*_^2^
Warning	62.77	< 0.001	0.060
Source Credibility	113.68	< 0.001	0.103
Warning × Source Credibility	12.98	< 0.001	0.013

#### Perceptions of deepfake video

**Fig. 4:**
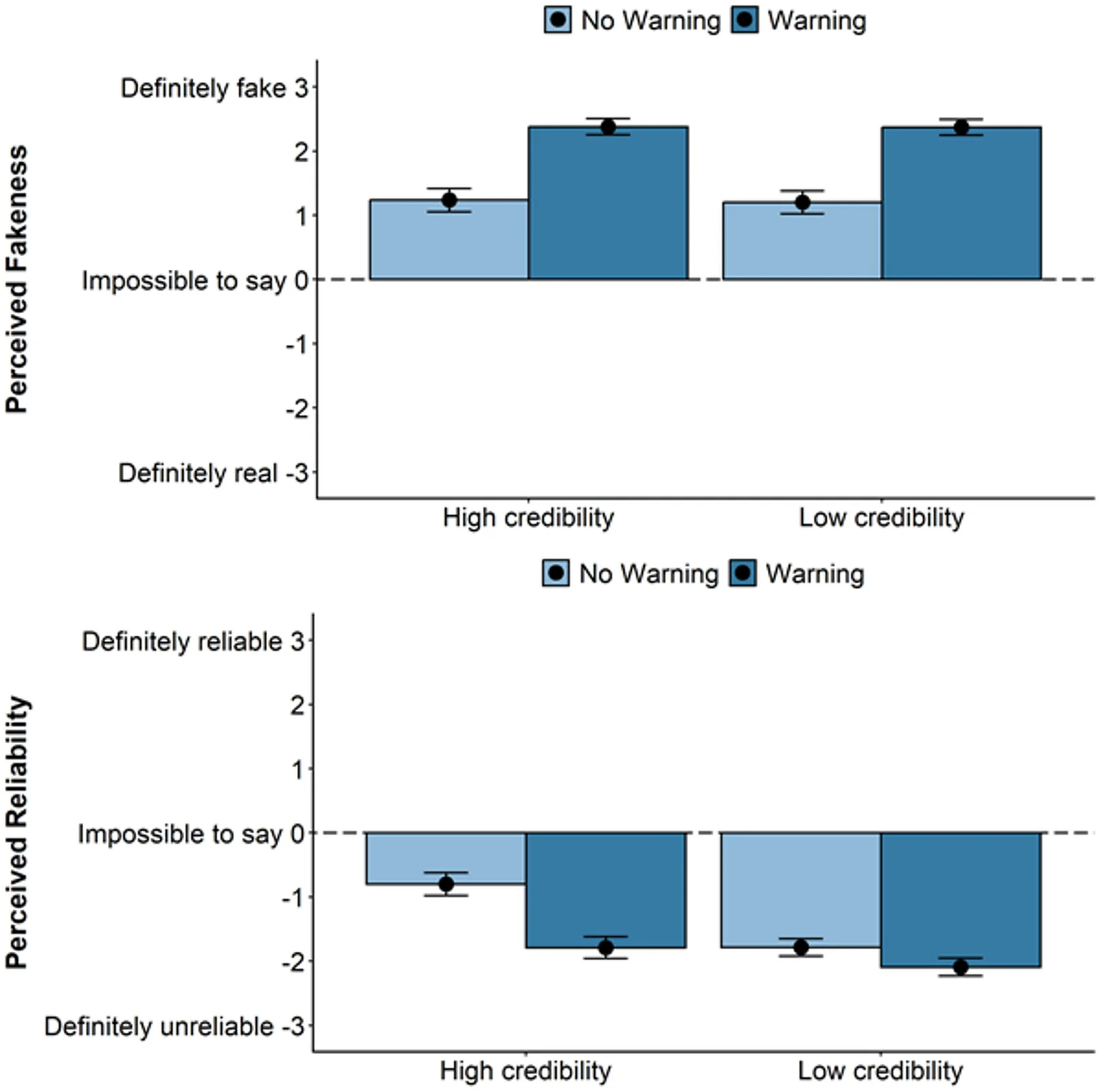
Mean Perceived Fakeness (top) and Reliability (bottom) of the Deepfake Video in *Experiment 2*. *Note.* Error bars denote 95% CIs.

To assess whether the warning or the alleged witness’s credibility influenced participants’ perceptions of the video, we again conducted two-way ANOVAs separately for perceptions of fakeness and reliability (Table 4). Consistent with Experiment 1, the warning significantly increased ratings of the video’s fakeness and reduced its perceived reliability, supporting H6a and H7a, respectively (Fig. 4). There was a significant main effect of source credibility on reliability scores, but not perceived fakeness; participants rated the video as providing more reliable information—but as no less likely to be a deepfake (contrary to H6b)—when the witness was presented as highly credible than when they were not, supporting H7b. The effect of source credibility on reliability significantly interacted with the effect of warning; the warning reduced the perceived reliability of the video regardless of the witness’s alleged credibility, but this reduction was larger in the high source credibility condition than in the low source credibility condition.

**Table 4:** Results of 2 × 2 ANOVAs on perceived deepfake fakeness and reliability in Experiment 2.

Effect	F(1, 992)	*p*	η_*p*_^2^
Fakeness			
Warning	216.88	< 0.001	0.179
Source Credibility	0.07	0.793	0.000
Warning × Source Credibility	0.03	0.858	0.000
Reliability			
Warning	65.84	< 0.001	0.062
Source Credibility	64.58	< 0.001	0.061
Warning × Source Credibility	18.16	< 0.001	0.018

#### Perceptions of the voiceover

Exploratory analyses revealed that participants rated the voiceover as less reliable when they had received a warning than when they had not; they also rated the voiceover as less reliable when it presented the witness as highly credible than when it undermined their credibility, but this difference was only significant in the warning condition (see the Supplementary Materials).

### Discussion

Experiment 2 replicated the finding that a specific warning is only partially effective at preventing the influence of a deepfake video on cognition. While explicitly undermining the witness’s credibility significantly reduced the perceived reliability of the deepfake accusatory video and its impact on perceived guilt to an extent, even the combination of a warning and a credibility-reducing voiceover was only able to diminish the deepfake effect on guilt perceptions by approximately half (relative to the no-warning, high source credibility condition). Finally, a specific warning influenced perceptions of source information, such that the voiceover was perceived as less reliable when it presented the witness as highly credible than when it undermined their credibility, and this difference was only significant in the warning condition.

## General discussion

In two experiments, we examined whether a specific warning could reduce the impact of a deepfake accusation video, and whether a warning was more effective when combined with other factors designed to undermine the witness’s credibility. Consistent with much prior research^6,14,16,42,72^, exposure to a deepfake video significantly influenced participants’ judgements; a specific warning that identified the video as fake decreased the perceived reliability of the video and increased its perceived fakeness, but only partially reduced its impact on guilt perceptions. Exposure to the deepfake produced higher guilt scores when the witness was presented with a foreign (Indian) accent than with a standard (British) accent—perhaps because of better deepfake detection with a familiar accent—and when the witness was presented as highly credible compared to when their credibility was undermined. However, combining a warning with a manipulation designed to undermine source credibility did not elicit stronger warning effects. Only the explicit source credibility manipulation of Experiment 2 significantly affected the warning’s impact, but in such a way that a warning produced a larger reduction in guilt judgements when the witness was presented as highly credible. Thus, manipulations designed to reduce source credibility did not make a warning more effective at reducing deepfake impacts, and a deepfake video continued to influence guilt perceptions even when the fake speaker was associated with low credibility and participants had received a specific deepfake warning.

The finding that the warning was effective at reducing the impact of the deepfake video across both experiments suggests that labelling AI-generated media may offer some utility for combatting misinformation, in line with prior research^32,41,42,73^. However, several caveats should be noted. First, warned participants continued to be influenced by the deepfake accusation in their judgements. This finding is consistent with prior work showing that while labels or warnings can reduce belief in misinformation, they seldom mitigate its influence entirely^43–46,74^. The continued influence of the deepfake in this study is somewhat surprising, however, given that corrective information often eliminates the impact of person-related misinformation (e.g., accusations) on person impressions^75,76^. One possible explanation for this discrepancy is that research on person impressions has typically used multiple untrue behavioural descriptions with few concrete elements, whereas the present study used an allegation referring to wrongdoing in a specific incident. This interpretation is broadly supported by theories of the continued influence effect, which suggest that corrections can leave gaps in people’s mental models of events; such gaps create cognitive discomfort^77,78^, motivating people to use any available information to complete their mental event model, regardless of its accuracy^45^. Applied to the present study, these theories suggest that retraction of misinformation relating to a person’s involvement in an event may have created a gap in participants’ mental models that was difficult to resolve in the absence of an alternative explanation, such that they were motivated to continue to rely on the deepfake accusation in their judgements.

Second, caution is warranted regarding the practical usefulness of specific warnings because they might also affect belief in subsequently presented (real) content. There is research to suggest that labelling some misleading content can lead people to assume that unlabelled (mis)information is accurate (i.e., the implied truth effect)^40^. Other studies, however, indicate that warnings about AI-generated content may instead reduce trust in both true and false information^31,35,36,79,80^; in line with this idea, supplementary analyses revealed that warned participants perceived source information—provided in the form of a voiceover—accompanying the deepfake as less reliable than unwarned participants. It should be noted, however, that participants in this study were only exposed to a single deepfake video, so further work is needed to elucidate how specific warnings impact people’s perceptions of subsequently presented media.

Our findings also provide further insight into how source credibility shapes the influence of misinformation. Specifically, Experiment 2 participants gave lower guilt judgements and perceived the deepfake as less reliable when the accompanying voiceover undermined (rather than bolstered) the alleged witness’s credibility, but nevertheless relied on the deepfake in their judgements to some extent. From a theoretical perspective, these findings are consistent with theories of the continued influence effect that highlight the importance of perceived information reliability in belief formation and updating^81^, as well as prior work showing that people are more likely to update their beliefs when information is provided by a relatively trustworthy source^49,50,54,82,83^. From a practical perspective, our findings indicate that discrediting a source can reduce the impact of misleading content, but may need to be combined with other interventions to eliminate misinformation influence entirely^29,55,84^. It should be noted, however, that the present study focused solely on the credibility of the person depicted in the deepfake; future research should therefore examine whether other types of source discreditation (e.g., targeting the creator of misleading content) can enhance the effectiveness of deepfake warnings.

Given concerns that deepfakes may be used to erode public trust in credible figures, the fact that a warning was more effective when the source was presented as highly credible in Experiment 2 provides some reason for optimism. This finding suggests that warnings can help to reduce the influence of deepfakes depicting trusted figures, which may be more influential than those depicting relatively unreputable characters if their artificial nature goes undetected. At the same time, the modest effect of a warning also provides further evidence that alerting users to deepfake videos is unlikely to neutralise their influence entirely. It is possible, however, that warnings may be more effective in cases where a deepfake clearly contradicts prior statements or actions of a depicted person who is well-known^72^. Future research could directly test if warnings are more effective when accompanied by information contradicting deepfake content (e.g., pointing out inconsistencies with the depicted person’s views)^85^ and could also examine whether warnings are more effective when they are accompanied by an explanation of the possible motive for creating the deepfake or how it was detected.

Our findings should be interpreted in light of some limitations. First, the warning in this study was presented in isolation for a minimum of five seconds prior to the deepfake, whereas warnings in real-world contexts are often subtle and may go unnoticed, meaning that they might be less effective than our findings suggest. To maximise warning effectiveness, it may be important to amplify the visibility of labels to ensure that users are alerted to the potentially dubious nature of the content. Second, the perceived fakeness and reliability of the deepfake were assessed through direct, single-item measures which may lack reliability^86^ and could have prompted participants to infer the expected response, thereby overestimating the extent to which the video was recognised as a deepfake. Third, deepfake labels are likely to be more challenging to implement in practice than in experimental research. While this study used a known deepfake, such fakeries are frequently difficult to identify in real-life contexts, given that both humans and algorithms struggle to detect synthetic or doctored media^3,7,87–90^. Fourth, source discreditation raises significant ethical concerns; for example, it may cause unjustified reputational harm when negative character judgements are attributed to depicted individuals in relation to things they never said or did. Such concerns are pertinent when deepfakes depict public figures, as discrediting the source could undermine people’s belief in all information the source provides, regardless of its accuracy^49,84^. It should be noted, however, that the purpose of this study was not to identify new methods for combatting deepfakes, but to further elucidate the cognitive processing of warnings, which are commonly used in real-world contexts in an attempt to reduce the impact of synthetic content. The findings should therefore be interpreted as evidence that credibility cues can shape the processing of deepfake content, rather than as support for interventions that target or undermine individuals.

To conclude, our findings suggest that both pre-emptive, specific warnings and directly discrediting the depicted source could help mitigate the influence of deepfakes. Both countermeasures reduced the perceived reliability of the deepfake and—to some extent—its impact on guilt judgements, suggesting they may have some utility for combatting the influence of synthetic media. Presenting the witness with a foreign Indian (vs. standard British) accent, however, did not reduce deepfake impact; rather, the Indian-accented deepfake was perceived as more reliable and elicited higher guilt judgements than the British-accented deepfake, suggesting that markers of AI-generated videos may be easier to detect when the receiver is relatively familiar with the accent used. Finally, our findings suggest that undermining source credibility is unlikely to be beneficial for increasing the effectiveness of deepfake warnings. In sum, while specific warnings are promising countermeasures to misleading AI-generated media, eradicating deepfake influences on cognition remains a substantial challenge.

## Supplementary Information

Below is the link to the electronic supplementary material.

Supplementary Material 1

## Data Availability

The data and materials for this study are available at: https://osf.io/5jx3h/overview? view_only=84a2f527811a4e15bf1c73f1fda1df99.
