# Supplementary material for: Credibility attacks do not enhance the impact of deepfake warnings
Source: Sci Rep. 2026 Aug 5;16:36702. doi: 10.1038/s41598-026-64243-y (PMC13727678; doi:10.1038/s41598-026-64243-y)
Supplement: Supplementary file 1 [file 41598_2026_64243_MOESM1_ESM.docx]

**Supplementary Materials**

**Incident Briefing**

Last Monday at 6:15am, a hit-and-run car crash occurred on the intersection of Richard Street and Victoria Lane in Clifton. The victim, Simone Brown (41) was turning out of Victoria Lane when a white sedan collided into the driver’s side of her vehicle. The incident resulted in serious harm to Brown, who suffered from fractured ribs, a dislocated shoulder, and a head injury. She was attended to by first responders and is currently recovering in hospital. Authorities are still searching for the driver of the white car, who fled the scene.

Eyewitness Statement: Paolo Rossi (18) It was…early morning. Maybe half-five? I was cycling down Victoria Lane on my way to work. I was meant to be on the opening shift. My headphones were in, so I didn’t hear anything, but when I turned onto Richard Street I saw there had been a crash and a white sedan was just speeding away. That driver just left the injured woman there alone. I didn’t know what to do! I couldn’t open the car door at first, and I called 999 when I realised she was unconscious. Thankfully, I got her awake and responding. The ambulance came not long after, but the police took a lot longer.

Impact Statement: Collette Brown (32), sister of the victim This has been a very difficult time for our family. Simone’s injuries are quite serious, and she’ll be recovering for a long time yet. It was a shock for everyone; I still vividly remember feeling frozen on the other end of the phone finding out what happened to her. I’ve been looking after the kids. I take them to visit her, but they are so young, it’s hard for them to see her like that. We’re all just doing our best to keep going. It is unfathomable how someone could hurt another person like that and then just drive off. I really hope they are caught.

Public Statement: Christian Whittaker (47), District Council member Having just learnt about another terrible accident on Richard Street in my hometown of Clifton, it must be said that there have just been too many incidents on this road. It’s dangerous! Terrible visibility, covered in potholes—it’s not good enough, and the district needs to do something about it. My sympathies go out to Ms Brown and her family. I assure you, the perpetrator will be caught and punished appropriately.

**Deepfake Script**

Well, I drive to work every day, so I wouldn’t know. Speaking of which, you know that crash that happened last week in Richard Street? It’s been on the news. Yeah, awful stuff. Well, I was actually there, just around the corner. Heard it and everything. Big awful crash, sounded like something out of a movie! Seriously. And you know how they’re still looking for the guy who fled the scene? Well, obviously I jumped out of my car when I heard a bang, went over to see what was happening, and got there just in time to see the driver pull out and speed off past me. His windows were tinted, so you couldn’t see from far away, but he drove right past me and I saw in, clear as day. It was Christian Whittaker. Yeah, the politician. I recognised him from those posters, a hundred percent. No doubt about it. He was the driver who hit the car.

**Voiceover Script for Experiment 2**

**High credibility:**
The following video has been circulating widely on social media and contains potentially relevant information regarding the ongoing hit-and-run investigation. It features *Dr Anjali Patel*, a long-time member of the community and respected local GP, who lives nearby and happened to be at the scene at the time of the incident. In the interview, recorded for a community podcast, she gives a composed and detailed account of what she witnessed, including information on the driver’s identity. Authorities are said to be reviewing the footage as part of their inquiries. Viewers should listen carefully to her account.

**Low credibility:**

The following video has been circulating widely on social media and contains potentially relevant information regarding the ongoing hit-and-run investigation. It shows a woman who says she saw the traffic incident. Sitting at what looks like her home office during a podcast interview, she makes an accusation against a member of the community. The alleged witness has been identified as *Anjalikumaraswamy Srinivasan*, a local socialite with a known record of seeking publicity and sharing strong opinions on community issues, who also has a number of historic fraud convictions. She does not appear to have any official connection to the investigation.

**Questionnaire in Experiment 1**

**Open-Ended Questions**

1. Consider the information you have been provided. Please summarise in 2-3 sentences what you think happened in Clifton on Monday morning.
2. What would be a good newspaper headline for the events of Monday morning?
3. Who should police speak to about the hit-and-run incident?

**Rating Questions**

The following questions were answered on a on a 7-point Likert scale ranging from -3 (*Definitely not*) to 3 (*Definitely yes*).

1. Are the roads in Clifton of good quality?
2. Did Simone Brown leave the crash without injury?
3. Have the police been effective in their handling of the situation?
4. Will police find the perpetrator of the hit-and-run incident?
5. Should police check for any existing CCTV footage near this intersection?

*Perceptions of Guilt*

6. Should Christian Whittaker be asked to resign from his position as District Councillor over the incident?
7. Was Christian Whittaker present at the scene of the crime?
8. Is Christian Whittaker guilty of a crime? (Follow-up, open-ended question: What were the reasons for your answer to the previous question?)

*Video Fakeness and Reliability*

9. Do you think the video was a deepfake?
10. Was the information provided by the video reliable?

**Questionnaire in Experiment 2**

**Rating Questions**

The following questions were answered on a on a 7-point Likert scale ranging from -3 (*Definitely not*) to 3 (*Definitely yes*).

** = Added for Experiment 2. All other questions were used in Experiment 1.

1. Are the roads in Clifton of good quality?
2. Did Simone Brown leave the crash without injury?
3. Have the police been effective in their handling of the situation?
4. Will police find the perpetrator of the hit-and-run incident?
5. Should police check for any existing CCTV footage near this intersection?

*Perceptions of Guilt*

6. Should Christian Whittaker be asked to resign from his position as District Councillor over the incident?
7. Was Christian Whittaker present at the scene of the crime?
8. Is Christian Whittaker guilty of a crime?
9. Was Christian Whittaker the driver who fled the scene?**

*Video Falsity and Reliability*

10. Do you think the video was a deepfake?
11. Was the information provided by the video reliable?

*Voiceover Reliability*

12. Was the information provided in the podcast clip reliable? **

**Descriptive Statistics**

**Table S1**
*Descriptive Statistics for Guilt and the Perceived Fakeness and Reliability of the Deepfake by Warning and Accent in Experiment 1*

| Condition | Guilt | |  | Fakeness | |  | Reliability | |
| --- | --- | --- | --- | --- | --- | --- | --- | --- |
|  | *M* | *SD* |  | *M* | *SD* |  | *M* | *SD* |
| Control | -2.51 | 0.44 |  |  |  |  |  |  |
| No Warning |  |  |  |  |  |  |  |  |
| British Accent | -0.33 | 1.38 |  | 0.94 | 1.50 |  | -0.58 | 1.36 |
| Indian Accent | 0.04 | 1.41 |  | 0.47 | 1.36 |  | -0.13 | 1.32 |
| Warning |  |  |  |  |  |  |  |  |
| British Accent | -1.28 | 1.29 |  | 2.42 | 0.91 |  | -1.67 | 1.43 |
| Indian Accent | -1.10 | 1.46 |  | 2.06 | 1.18 |  | -1.31 | 1.53 |

**Table S2**
*Descriptive Statistics for Guilt and the Perceived Fakeness and Reliability of the Deepfake by Warning and Speaker Credibility in Experiment 2*

| Condition | Guilt | |  | Fakeness | |  | Reliability | |
| --- | --- | --- | --- | --- | --- | --- | --- | --- |
|  | *M* | *SD* |  | *M* | *SD* |  | *M* | *SD* |
| Control | -2.12 | 0.85 |  |  |  |  |  |  |
| No Warning |  |  |  |  |  |  |  |  |
| High Credibility | 0.52 | 1.21 |  | 1.24 | 1.46 |  | -0.80 | 1.45 |
| Low Credibility | -0.58 | 1.07 |  | 1.20 | 1.43 |  | -1.78 | 1.09 |
| Warning |  |  |  |  |  |  |  |  |
| High Credibility | -0.37 | 1.45 |  | 2.38 | 1.01 |  | -1.79 | 1.35 |
| Low Credibility | -0.92 | 1.13 |  | 2.38 | 0.99 |  | -2.09 | 1.12 |

**Table S3**
*Means and Standard Deviations of Perceived Voiceover Reliability in Experiment 2*

| Condition | Voiceover Reliability | |
| --- | --- | --- |
|  | *M* | *SD* |
| No Warning |  |  |
| High Credibility | -0.25 | 1.51 |
| Low Credibility | -0.35 | 1.59 |
| Warning |  |  |
| High Credibility | -1.10 | 1.59 |
| Low Credibility | -0.55 | 1.82 |

**Analysis on Perceived Voiceover Reliability**

We conducted an exploratory 2 (warning: no warning, warning) × 2 (source credibility: high, low) ANOVA to examine whether the warning affected the perceived credibility of the voiceover introducing the witness. There were significant effects of warning and source credibility, as well as a significant interaction (Table S4). Participants rated the information provided by the voiceover as less reliable when they had received a warning than when they had not; they also rated the voiceover as less reliable when it presented the witness as highly credible than when it undermined their credibility, but this difference was only significant in the warning condition (no warning: *F*[1, 992] = 0.48, *p* = .491, η_p_^2^ = 0.001; warning: *F*[1, 992] = 14.05, *p* < .001, η_p_^2^ = 0.015; see Figure S1). In sum, these findings indicate that specific warnings may reduce the perceived credibility of source information accompanying a deepfake, particularly when it presents the depicted person as highly credible.

**Figure S1**

*Mean Perceived Reliability of the Voiceover in Experiment 2*

*Note.* Error bars denote 95% CIs.

**Table S4**
*Results of 2 × 2 ANOVAs on Perceived Reliability of the Voiceover in Experiment 2*

| Effect | *F*(1, 992) | *p* | η_p_^2^ |
| --- | --- | --- | --- |
| Warning | 25.96 | < .001 | 0.026 |
| Source Credibility | 4.69 | .031 | 0.005 |
| Warning × Source Credibility | 9.86 | .002 | 0.010 |
